# Water Uptake by Evaporating pMDI Aerosol Prior to Inhalation Affects Both Regional and Total Deposition in the Respiratory System

**DOI:** 10.3390/pharmaceutics13070941

**Published:** 2021-06-24

**Authors:** Victoria Legh-Land, Allen E. Haddrell, David Lewis, Darragh Murnane, Jonathan P. Reid

**Affiliations:** 1 School of Life and Medical Sciences, University of Hertfordshire, Hatfield AL10 9AB, UK; v.legh-land@herts.ac.uk (V.L.-L.); d.murnane@herts.ac.uk (D.M.); 2 School of Chemistry, University of Bristol, Bristol BS8 1TS, UK; a.haddrell@bristol.ac.uk; 3 Chiesi Farmaceutici S.p.A, Chippenham SN14 0AB, UK; d.lewis@chiesi.com

**Keywords:** metered dose inhaler, spray plume aging, water condensation, aerosol hygroscopic growth, deposition modelling

## Abstract

As pulmonary drug deposition is a function of aerosol particle size distribution, it is critical that the dynamics of particle formation and maturation in pMDI sprays in the interim between generation and inhalation are fully understood. This paper presents an approach to measure the evaporative and condensational fluxes of volatile components and water from and to solution pMDI droplets following generation using a novel technique referred to as the Single Particle Electrodynamic Lung (SPEL). In doing so, evaporating aerosol droplets are shown capable of acting as condensation nuclei for water. Indeed, we show that the rapid vaporisation of volatile components from a volatile droplet is directly correlated to the volume of water taken up by condensation. Furthermore, a significant volume of water is shown to condense on droplets of a model pMDI formulation (hydrofluoroalkane (HFA), ethanol and glycerol) during evaporative droplet ageing, displaying a dramatic shift from a core composition of a volatile species to that of predominantly water (non-volatile glycerol remained in this case). This yields a droplet with a water activity of 0.98 at the instance of inhalation. The implications of these results on regional and total pulmonary drug deposition are explored using the International Commission of Radiological Protection (ICRP) deposition model, with an integrated semi-analytical treatment of hygroscopic growth. Through this, droplets with water activity of 0.98 upon inhalation are shown to produce markedly different dose deposition profiles to those with lower water activities at the point of inspiration.

## 1. Introduction

Pressurised metered dose inhalers (pMDIs) are widely used in the treatment of respiratory conditions [1] and are typically formulated with a liquefied propellant (hydrofluoroalkane (HFA) in modern pMDI products), containing either a colloidal suspension of micronized drug or drug in solution [2,3]. Formulations may also contain volatile solvents (usually ethanol) or non-volatile excipients (e.g., glycerol, povidone or macrogol 1000) to aid solubility [3,4] or modify the aerodynamic diameter of the emitted aerosol droplets [5,6]. The effectiveness of a pMDI is directly dependent on its capability to produce drug aerosol particles that reach, and are deposited in, the target region of the respiratory system to instigate therapeutic effect [7]. It is generally accepted that for effective pulmonary deposition, aerosol particle sizes must be 1–5 µm in diameter: larger particles will impact in the oropharynx and smaller particles have insufficient inertia for deposition [8]. Consequently, there has been significant research into the accurate measurement and prediction of pMDI aerosol generation and sizing. However, the physical process of aerosolization from pMDI formulations is extremely complex and a comprehensive understanding is yet to be fully realised, in part due to the extremely short time and length scales on which droplet generation occurs, and the experimental difficulties in studying the transient, highly dense regions of spray [9].

What is known is that pMDI droplet formation occurs in two distinct phases: primary atomisation and secondary ageing processes. There has been extensive research into the atomisation phase of droplet production by pMDIs and, while the model’s exactitude remains elusive, the mechanism proposed by Clark et al. [10] is considered representative and shows good agreement with experimental data [9]. In this mechanism, the propellant rapidly expands and undergoes flash evaporation, resulting in the formulation forming a two-phase flow [2,11]. During the swift, subsequent expulsion from the actuator, the remaining liquefied component is dispersed into droplets through shear forces [10,12], and the initial droplets undergo further processes of evaporation (also known as droplet ‘ageing’). The resultant size of this initial droplet distribution has long been shown to be dependent on the formulation characteristics (such as viscosity, surface tension and non-volatile solute concentration) and device design [13,14,15,16,17,18,19]. The initial droplet diameters have been measured using laser diagnostic techniques [20,21,22,23], but have also been estimated through back-calculations from terminal aerosol size distributions by assuming a simple model of evaporation kinetics [24,25,26,27].

**Figure 1:**
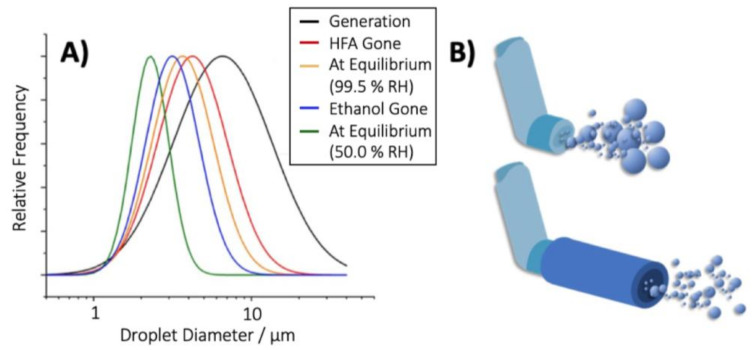
Schematic representation of the evolution of the size distribution for an idealised pMDI spray. (**A**) The change in droplet size distribution at different phases of aerosol ageing following initial generation. (**B**) Attaching a spacer to the pMDI provides sufficient time for droplet ageing to occur, such that the large droplets of pMDI sprays evaporate to form an aerosol with a smaller, narrower size distribution.

During spray emission, the evolution of the spray’s size distribution is driven by the change of aerosol droplet momentum, and the heat and mass flux with the surrounding environment [9]. As the aerosol plume proceeds from the actuator orifice, energy attained from the ambient environment provides sufficient latent heat of vaporisation to induce the evaporation of the remaining liquefied volatile species. In the case of a mixed species droplet, the more volatile components evaporate preferentially and the presence of non-volatile excipients does not impede vaporisation. Therefore, for pMDI formulations, the following evaporative distillation sequence is proposed: firstly, the HFA propellant evaporates followed by the ethanol co-solvent and any other volatile compounds present [28]. Overall, these evaporative processes result in a shift of the droplet size distribution (Figure 1A) and the thermal cooling of the droplet [29]. It is this ‘aged’ aerosol droplet which will be inhaled by the patient, and therefore the physics of droplet ageing directly affect the aerodynamic diameter and droplet deposition in the respiratory system.

There remains debate, within the limited literature examining the topic, over the extent that relative humidity (RH) affects the secondary ageing of pMDI aerosol plumes. It has been suggested that the rapid evaporation and cooling of pMDI droplets would enable them to act as condensation nuclei for water [30]. Initially this hypothesis was disregarded as studies showed humidity to have no significant effect on solution droplet distributions [31,32]. However, more recent work revealed that droplet distributions generated into raised humidity environments displayed substantial increases in the mass median aerodynamic diameter (MMAD) compared to those produced in lower ambient humidities [33]. Further research comparing these studies suggested that the discrepancies may have arisen from the differences in elapsed time between aerosol generation and characterisation, with the earlier works leaving longer time periods between sample collection and analysis [30]. As such, it was suggested that transient condensational growth may occur, but be short-lived, with water relinquished back to the surroundings as the aerosol quickly equilibrates to the ambient conditions [30,34]. This appears to be corroborated by a recent single droplet analysis of ethanol which reported the condensation of water in a humid environment to be concurrent with the evaporation of ethanol [35]. Furthermore, the study revealed the capacity for the complete conversion of an ethanol droplet to that of a pure water droplet on extremely short timescales (of order 1 s).

The simultaneous condensation of water during the evaporative ageing of pMDI aerosol plumes could have significant implications for the inhaled droplet size distribution and subsequent pulmonary deposition, but further investigation is required into its generality for pMDI drug formulations. Recent studies have developed models for the empirical inference of the initial droplet diameters from their residual impactor determined distributions [25]. However, these models are based on simple evaporation kinetics as well as the assumption that volatile species are fully evaporated upon entry into an impactor. Such models show reasonable agreement with laser diagnostic measurements for early-phase droplets, but differences in the measured MMAD and distribution are common and are extremely sensitive to formulation and the axial distance of the laser measurement [36,37,38].

To complicate the measurement of aerodynamic size distributions from pMDI products further, pMDI aerosols display a substantial ballistic deposition effect in impactor inlets (or the oropharynx during inhalation). Droplets which possess a size suitable for collection on the classification stages of cascade impactors are deposited on the induction port due to the ballistic nature of the high velocity pMDI spray. This ballistic fraction is therefore excluded from determination of the size distribution of the aerosol as emitted from the pMDI. A spacer chamber is often used to reduce the oropharyngeal deposition from pMDIs. Ideally, a spacer should only alter the aerosol plume and subsequent deposition by removing the ballistic fraction, leaving the pulmonary fraction unchanged (Figure 1B). In most cases, spacers are associated with a substantial reduction in large particles impacting in induction ports and the fine particle fraction (FPF) remains unchanged or is occasionally increased [39,40]. The slight increase in FPF is hypothesised to be the result of the longer evaporation period the droplets experience, and the ensuing smaller droplets at the point of inhalation [41]. However, pMDI droplets dispensed into a spacer experience vastly different conditions to those sprayed directly into the mouth. As such, further research is required on the effect on aerosol dynamics within spacers prior to inhalation, with a particular focus on HFA formulations and the effect increased droplet residence time has on particle size.

To optimise drug deposition and patient outcomes, the aerosol dynamics of droplet formation from pMDI sprays need to be fully understood to allow for the prediction of aerosol size distributions, and their transition, from generation to deposition. As such, in this paper the effect that the rapid evaporation of propellant, and the potential resulting water accretion, has on the aerosol plume during the actuation of an pMDI inhaler is explored. Initially, the dynamics of aerosol produced from pMDI starting formulations are measured using novel single droplet analysis instrumentation. We then employ the International Commission on Radiological Protection (ICRP) deposition model with an incorporated, semi-analytical treatment of hygroscopic growth. In doing so, we begin to explore the potential ramifications that this enhanced understanding of the evolution of the aerosol properties of pMDI sprays may have on predicted regional dose deposition in the respiratory system.

## 2. Experimental

### 2.1. The Single Particle Electrodynamic Lung (SPEL)

**Figure 2:**
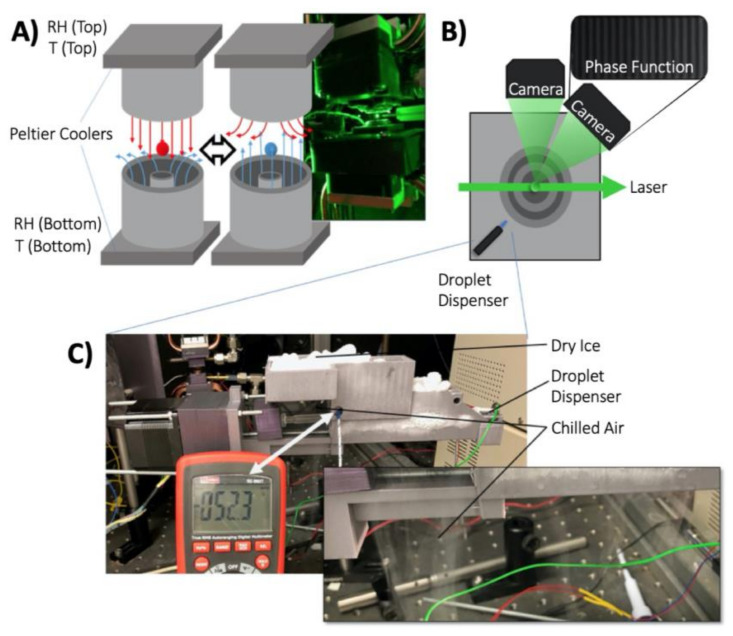
Experimental setup used to measure the rapid evaporation of droplets originally consisting of volatile solvents. (**A**) Schematic of the electrode orientation and air flow directions used to levitate and condition an individual droplet. The inset photo shows an individual droplet (radius < 10 µm) trapped by the electrodynamic field and illuminated by the laser. (**B**) Top-down view of the levitation region of the SPEL, indicating the orientation of the droplet dispenser, laser, and cameras used to position and collect the phase function of the levitated droplet. The concentric circles indicate the relative position of the high voltage and ground electrodes. (**C**) Photograph of the syringe pump used to cool the syringe and droplet dispenser used to produce individual HFA droplets that can be injected into the SPEL. The inset photo shows the cool vapour encompassing the region holding the HFA loaded syringe, which maintains the temperature between −50 and −55 °C.

The ability to probe the physicochemical properties of an individual droplet trapped in an electrodynamic field has long been reported [42,43]. Depending on the orientation of the electrodes, various properties of a trapped droplet can be measured, including the radius, mass, chemical composition, and droplet or particle structure [44,45]. The Single Particle Electrodynamic Lung (SPEL) introduced here utilises a pair of concentric cylinder electrodes to trap the droplet in confined space (Figure 2A), in which the temperature and relative humidity of the air flow that encompasses the levitated droplet is highly controlled. The SPEL is based on the comparative kinetic electrodynamic balance (CK-EDB [46]) but has many notable advances, including its open-air design and asymmetric temperature controls (Figure 2A). Among the many features that these modifications offer the end user is the ability to expose the droplet to RHs above 100%, a supersaturated gas phase as utilised in this study.

Air flows were introduced to the device through both the top and bottom electrodes (volumetric flow rates of either 20 or 200 mL/min), with the droplet exposed to the RH and temperature of the air with the greater flow rate (200 mL/min). The temperature of both electrodes was independently controlled by Peltier coolers, establishing a temperature gradient across the region with the temperature of the top electrode set to a temperature much lower than the bottom electrode (e.g., 0 °C vs. 20 °C). This temperature gradient between the electrodes (positioned 4 mm apart) ensured that the RH of the air flow passing through the bottom electrode at a RH ~90% (at a flow rate of 200 mL/min) surpassed supersaturation (RH >100%) within the region of a trapped droplet. To confirm this, the temperature and RH in the trapping region were inferred by analysing the evaporation profile of an individual droplet (NaCl solution) of known initial salt concentration.

A droplet on demand dispenser (Microfab, Plano, TX, USA) was used to produce and target an individual droplet comprising of HFA (provided by Chiesi Farmaceutici S.p.A, Chippenham, UK), ethanol and glycerol (Sigma Aldrich, Gillingham, UK) into the levitation region of the SPEL (Figure 2B). The presence of an induction electrode positioned ~1 mm from the tip of the dispenser (not shown) induced a slight net charge on the droplet during generation, ensuring routine trapping of a droplet within the electrodynamic fields produced by the concentric cylinder electrodes (Figure 2A,B). The exact time of droplet production was known and ~0.1 s were required for the droplet to travel from the point of generation to the point of stable trapping in the electrodynamic field. Once trapped, the size and structure of the droplet were inferred from the interpretation of the angularly resolved elastic light scattering pattern, referred to as the phase function (collected at a frequency ~100 Hz), produced by the scattering of the laser light from the trapped droplet. Through altering the frequency of the electrodynamic fields, the rapid mass change of the evaporating droplet was accounted for and enabled the droplet to remain in the centre of the trap throughout the entire dynamic process.

The dominant component of the starting formulation of pMDI droplets is a highly volatile species, such as HFA, which has a boiling point of −25 °C. Thus, the entire droplet dispenser assembly, consisting of the droplet dispenser and a glass 1 mL syringe, was cooled to −50 to −60 °C with dry ice (Figure 2C). This allowed for the precise and reproducible production and targeting of individual droplets, consisting of highly volatile species, into the SPEL setup with a well-defined generation time point.

### 2.2. ICRP Model with Specific Hygroscopic Growth

**Figure 3:**
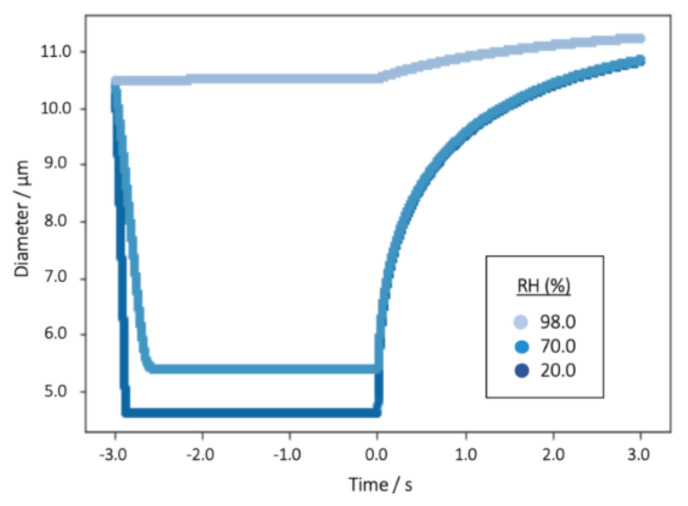
The equilibration of a single 10.5 µm diameter glycerol–water solution droplet (initial water activity of the droplets, derived from the starting solution concentration of 10% *w*/*v*, is set at 0.98), through the evaporation of water, into three RH environments (20, 70 or 98%) before subsequent growth in 99.5% RH. The 3.0 s holding time in the model was used to ensure that the water activity of the aerosol at the point of inhalation (*t* = 0 s) was stable at the desired value.

The ICRP model is a semi-empirical model that was developed for radiological protection purposes, but is now widely accepted as a standard for whole lung dosimetry calculations [47]. While the ICRP guidelines provide a method to account for the hygroscopic growth of droplets, previous publications have shown them to be simplistic and they significantly underestimate particle growth, and thus deposition [48]. In comparison, former work has provided extensive verification of the semi-analytic solution to the heat and mass transfer equations provided by Kulmala et al. [49] for the hygroscopic growth of droplets [50,51,52]. Consequently, this treatment of droplet hygroscopic growth was integrated into the ICRP model to allow the hygroscopic behaviour of the aerosol plume during inhalation to be accurately incorporated into the deposition modelling. This incorporated model has been described in detail in earlier work and shown to yield deposition profiles that show excellent agreement with experimental studies [48]. However, it should also be noted that, in cases of significant changes in droplet temperature from the ambient during droplet growth or evaporation (>3 °C), the accuracy of the Kulmala model is reduced (e.g., the rate of mass flux from the droplet equilibration modelled at 20% RH in Figure 3 will be marginally higher than that observed experimentally). As both the ICRP model and the hygroscopic growth treatment have been comprehensively described in former publications [47,49,53,54,55], only a summary of the model methodology will be presented here. A summary of all abbreviations and parameters used in the subsequent equations are also provided in Table 1 and Table 2, respectively.

Due to the complexity of HFA and ethanol evaporation, and results from the SPEL experiments (explored in further detail later in the paper), the hygroscopic treatment model utilised here considers only the evaporation and condensation of water onto glycerol solution droplets in the prediction of droplet evaporation and hygroscopic growth prior to and upon inhalation into the respiratory system. A log-normal distribution for the initial droplet sizes glycerol–water solution aerosol plume, of a known concentration, generated from the pMDI, was calculated from the literature [24,56] (glycerol concentration 10% *w*/*v*, mass median diameter (MMD) of 10.5 μm, geometric standard deviation (GSD) of 1.8). A range of equally spaced droplet log-diameters that fully encompassed the distribution were then identified. This series of initial starting sizes was then run simultaneously through the hygroscopic growth treatment and ICRP model to calculate the deposition of glycerol–water solution droplets from a model pMDI aerosol plume.

The radial growth factor, *GF_r_* of an aerosol particle containing an involatile solute at a specified relative humidity, RH, is given by:

(1) $$GF_{r}=\frac{r\left(RH\right)}{r\left(RH=0\right)}$$

where *r(RH)* is the radius of the wet droplet at a specific RH and *r(RH = 0)* is the dry particle radius at 0% RH. Provided that the density of both the solution and dry solute particle is known (in this experiment a glycerol concentration of 10% *w/v* was used), the radial growth factor can be converted simply into a mass or volumetric growth factor.

As solution pMDI formulations are assumed to be homogeneous, and thus the initial solute concentrations of all droplets in the distribution are equivalent, the model used the known initial solute concentration, density, and radius of a sample glycerol–water solution droplet to calculate its mass fraction of solute and radial growth factor following the complete evaporation of HFA and ethanol. By then comparing the modelled droplet growth factor to a calibration curve of growth factors against water activity, generated using the hygroscopicity data of glycerol [57], the initial water activity of all the glycerol–water solution droplets within the model distribution was determined. Similarly, the initial density of the droplet was ascertained using a calibration curve for density against water activity. From a starting water activity (0.98), based on the initial concentration of the glycerol–water solution that will be determined from our measurements and is justified below, the mass flux of water, *I* (g s^−1^), to or from each droplet in the distribution in an environment of either 20, 70 or 98% RH was calculated using Equation (2):

(2) $$I=-4\pi a\frac{S_{\infty }-a_{w}}{\frac{RT_{\infty }}{M\beta_{M}ADp_{v,\infty }}+\frac{a_{w}L^{2}M}{R\beta_{T}KT_{\infty }^{2}}}\left(\frac{Sh}{2}\right)$$

where *a* is the droplet radius, *S_∞_* is the saturation ratio of water in the gas phase away from the droplet surface and *a_w_* is the water activity at the droplet surface. *R* is the gas constant; *T_∞_* is the temperature of gas phase; *M* is the molar mass of water; *A* is the Stefan flow correction factor; *D* is the diffusion coefficient of water in the gas phase (in this case, a combination of water vapour and air) and *p_v,∞_* is the saturation vapour pressure of water. *L* is the latent heat of vaporisation of water; *K* is the thermal conductivity of the carrier gas; *β_M_* and *β_T_* are the Fuchs–Sutugin transitional correction factors for mass and heat transfer respectively and, finally, *Sh* is the Sherwood number to correct for the influence of a moving gas phase on the mass transport kinetics. The mass and thermal accommodation coefficients were taken as unity and the droplets were assumed to remain homogeneously mixed and experience no bulk limitations to mass transport throughout (i.e., diffusional mixing transpires significantly more quickly than the transport of water).

Once computed, the mass flux of water was multiplied by the simulation time step (0.0001 s) to yield the change in mass and the new droplet volume, radius and density during a single step in the model iteration. The model was progressed for sufficient time to allow the droplets to fully equilibrate in a specified RH, of either 20, 70 or 98%, and at 293.15 K to ensure that the hygroscopic growth and deposition of droplets upon inhalation was modelled purely for aerosol for which the water activity at the point on inhalation was known, stable and equivalent throughout the entire droplet distribution. This was crucial in order to begin to explore the impact that the water activity of the pMDI droplets at the instance of inhalation has on their subsequent deposition pattern in the respiratory system. Furthermore, this equilibration period, which modelled the dynamics of droplets into similar conditions to those generated into a valved holding spacer chamber, allowed for the contribution of ballistic deposition of pMDI aerosols in the oropharyngeal region to be disregarded from the deposition modelling.

Equation (2) was then applied again to the aerosol droplets pre-equilibrated into either 20, 70 or 98 % RH prior to inhalation, but with conditions set to simulate the resultant hygroscopic growth of the glycerol droplets upon inspiration into the respiratory system (i.e., 310.15 K, 99.5 % RH [58]). This allowed for the evaporation and growth of the droplets following the complete loss of HFA and ethanol, but prior to and upon inhalation, to be observed (Figure 3). The model was used to estimate the radius of the droplet at times corresponding to the moment of departure from each of the regions in the lung in the ICRP model. These were then used to estimate the relative regional deposition efficiencies and fractions, as detailed in an earlier publication [48].

Particle deposition within the ICRP model is calculated by likening a single breath to a volume of particle-laden air passing through a series of filters. Each filter corresponds to one of four regions of the respiratory system: the extrathoracic region (ET), bronchial region (BB), bronchiolar region (bb) and the alveolar-interstitial region (AI). The deposition of droplets on each filter stage by either ‘aerodynamic’ (impaction) or ‘thermodynamic’ (sedimentation and diffusion) mechanisms are subject to the droplet’s size and the air flow rate. For each of these deposition mechanisms, the deposition efficiency (also filtration efficiency), *η,* of particles in each region is then given by:

(3) $$\eta =1-e^{-a_{ICRP}\;R_{ICRP}^{p}}$$

where *a_ICRP_*, *R_ICRP_* and *p* are constants specific to the region in the respiratory system and deposition mechanism. For particles where both deposition methods are prevalent, the total deposition efficiency can be obtained through the quadratic sum:

(4) $$\eta_{Total}=\sqrt{\eta_{ae}^{2}+\eta_{th}^{2}}$$

where *η_Total_* is the total deposition efficiency of a particular respiratory region and *η_ae_* and *η_th_* are the respective aerodynamic and thermodynamic deposition efficiencies for that region. Once the combined deposition efficiency is computed, the deposition fraction, DE, for each region can be determined by:

(5) $$DE_{j}=DE_{j-1}\eta_{j}\frac{\phi_{j}}{\phi_{j-1}}\left(\frac{1}{\eta_{j-1}}-1\right),\;for\;j=1,\;N$$

where *η_j_* is the total deposition efficiency for region, *j*, *ϕ_j_* is the volumetric fraction for region j and N is the number of regions. It should be noted that the BB and bb deposition are commonly summed to yield a total bronchial deposition value (BR), and indeed that is the case in this model. Similarly, through the summation of the deposition fractions of all regions, the total deposited fraction can be ascertained.

By the adjustment *a_ICRP_*, *R_ICRP_* and *p,* the ICRP model is capable of accounting for subject specific differences such as height, sex, activity level, breathing rate and volumes. All simulations in this paper were calculated for a healthy, adult Caucasian female, who was sitting, with particles inhaled solely through mouth breathing. The exact values used for *a*_ICRP_, *R_ICRP_* and *p* and subject specific factors can be found in Supplementary Materials Tables S1 and S2.

The frequency of droplets for a specific starting diameter in an aerosol plume can be estimated from the integration of the starting log-normal distribution. Using this estimation and the known starting solute concentration, the mass of glycerol for all the droplets in the population was computed and a mass distribution of glycerol droplets determined. Through the coupling and summation of this mass distribution and the ICRP deposition data for the specific initial droplet diameters, which were tracked throughout the model, the total dose of glycerol deposited in each of the regions of the respiratory system was calculated.

## 3. Results

### 3.1. Evaporation Dynamics of Individual Droplets Consisting of Volatile Solvents

The rate at which an evaporating water droplet changes size is highly dependent on the RH: as the RH is increased the rate of evaporation will decrease (Figure 3). This relationship is well understood and readily predictable. Indeed, it is the origin of the requirement to cool inertial impactors when sizing liquid nebulizer products [59,60,61].

**Figure 4:**
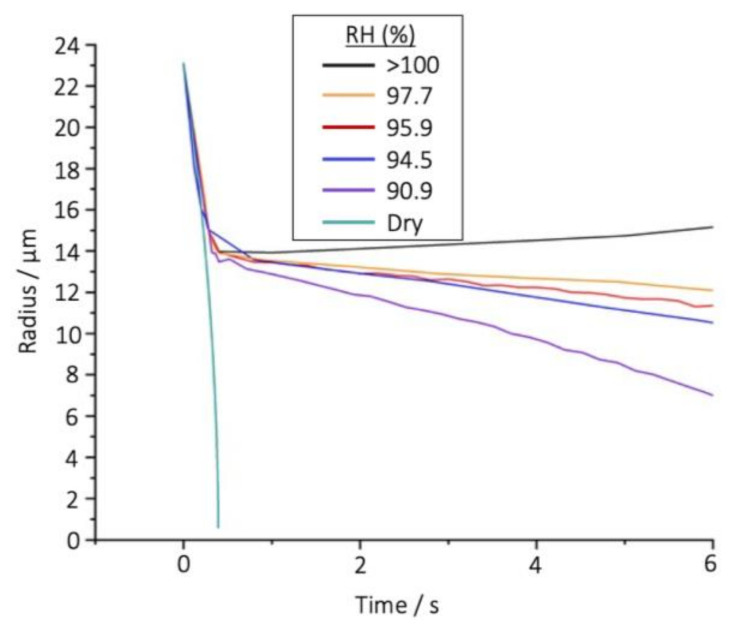
Evaporation of pure ethanol (experimental) droplets in an airflow of various RH. In each case, the evaporation rate of a pure water probe droplet was used to estimate the RH following the method of Davies [62].

The evaporation profile of droplets of a volatile species, such as pure ethanol, is affected by RH in a markedly unique way when compared with water droplets (Figure 4). The fast evaporation of the ethanol results in considerable cooling and a reduction in droplet temperature by up to 17 °C; in turn, this substantial lowering of temperature causes the rapid condensation of water from the gas phase onto the droplet [35]. This converts the droplet from pure ethanol to pure water by the time the evaporation rate dramatically slows (~0.35 s). Prior to the abrupt change in flux, the droplet follows the evaporation dynamics of pure ethanol; however, following this change, the evaporation profile instead matches that of a water droplet. From these stark differences in the evaporation profiles it is clear that, after less than a second, the entire chemical composition of the droplet has transformed from pure ethanol to pure water, consistent with previous observations [35]. Notably, the time at which the mass transfer rate, and thus the gradient in radius with time, suddenly changes was found to be approximately independent of RH, occurring at around the same size in each RH case. Dramatically, this independence was found to even include conditions of gas phase supersaturation (i.e., above 100% RH). Thus, even in circumstances where there is an excess of water in the vapour phase, the amount of water added to the droplet itself is dependent on the mass/heat flux of the droplet during evaporation of ethanol.

**Figure 5:**
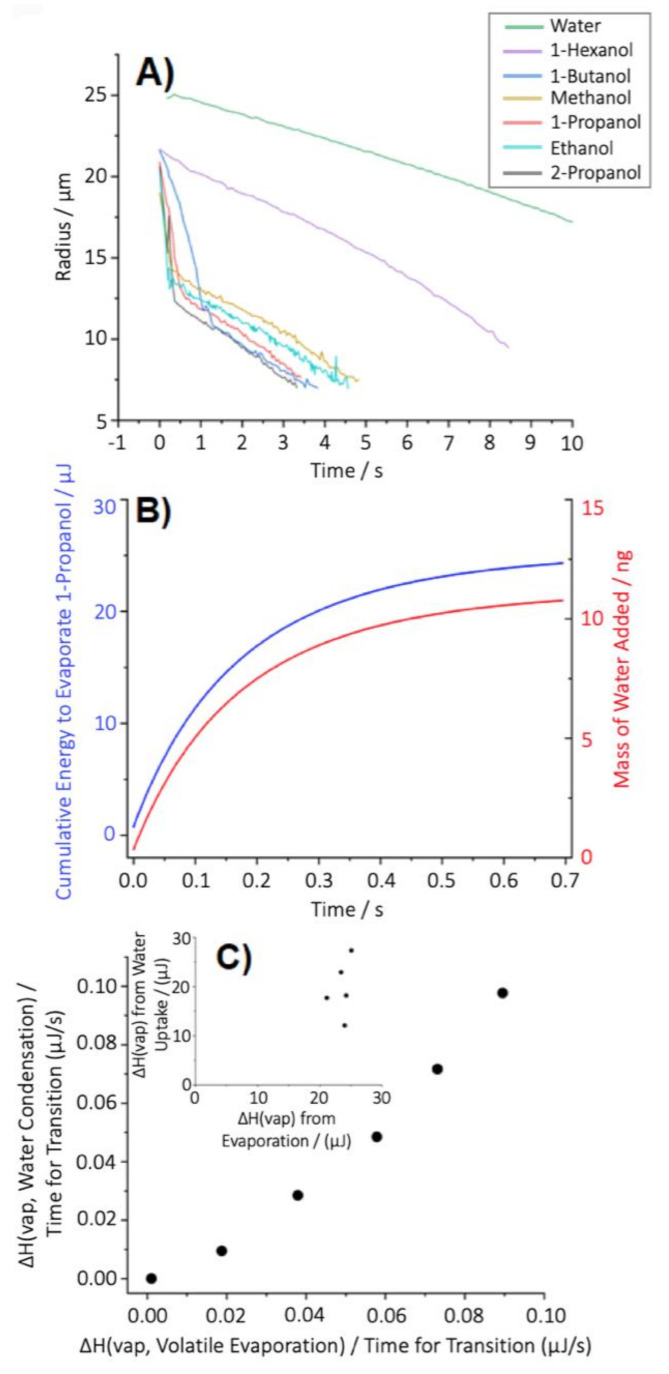
Exploring the underlying processes that govern the transition of a rapidly evaporating (volatile) droplet at elevated RH into a water droplet. (**A**) Evaporation measurements of individual droplets of various initial compositions in an air flow with an RH ~85 %. (**B**) Equivalence of the mass of water accumulated by a droplet if the vaporization enthalpy of 1-propanol from a droplet of initial radius of 21.3 µm was entirely balanced by the condensation energy of water. Ultimately, this would be equivalent to a pure water droplet with a radius of 14 µm and a mass of 11.4 ng. (**C**) The phenomenological relationship between the enthalpy required for evaporation of a volatile droplet and the enthalpy liberated on condensation of water. Both are normalised by the time during which the early dynamics change the droplet from a pure volatile to pure water droplet. When the time taken for the transformation is not considered (inset), the relationship between the enthalpies is not as clearly defined.

To further explore and better understand the transition profiles of evaporating volatile droplets that initially start as droplets of pure, volatile solvents, the evaporation dynamics of a range of volatile components of varying vapour pressure were measured into humid air (Figure 5). As the point of transition from an ethanol evaporation profile to a water evaporation profile was found to be independent of the ambient RH, subsequent experiments were carried out in a humid RH that allowed for the easy trapping of droplets within the experimental system. Consequently, individual droplets consisting of pure volatile solutions were injected into an air flow with an RH 85 ± 3%, and their radii measured over time (Figure 5A). As with ethanol, the dramatic shift from a rapidly drying droplet of a pure volatile species to a droplet of pure water was observed for almost all the species, with only the least volatile solvent not observed to convert entirely to a pure water droplet (1-hexanol).

As previously demonstrated with pure ethanol, the conversion of the composition of a rapidly evaporating droplet from a pure volatile species to a droplet that can be assumed as predominantly composed of water was found to be driven by the dramatic reduction in temperature of the droplet during evaporation and the accretion of water from the gas phase onto the cold droplet surface [35]. As an example, consider the evaporation of a 1-propanol droplet (Figure 5B). The cumulative enthalpy required to drive the evaporation of 1-propanol can be calculated as the measured droplet radius and, thus, mass change from knowledge of the molar enthalpy of vaporisation of liquid 1-propanol. The corresponding increase in water mass can be inferred if it is assumed that this heat loss is balanced by the condensation of water from the gas phase onto the droplet. This leads to an estimate of condensed water mass of 11.4 ng, equivalent to a droplet radius of 14 μm, marginally larger than the size of ~13 μm at the experimental time at which the evaporation rate markedly changes to be equivalent to that of a pure water droplet. Although providing an over-estimate, this simple accounting of mass and heat exchange provides a reasonably accurate estimate of the condensed water mass.

Estimating the conversion of volatile species to water during evaporation, based solely on the balancing of enthalpy lost by evaporation with enthalpy gained by condensation, was found to be largely consistent across all the volatile components considered (Figure 5C). Indeed, phenomenologically, the data in Figure 5C indicate that the correlation is even stronger when the time taken for the near complete loss of the volatile component is also accounted for, presumably related to the magnitude of the heat flux driving evaporative cooling. Identifying the specific time at which there is an abrupt change in the mass flux (Figure 5A) is limited by the time-uncertainties in both the initial droplet generation time (~50 ms) and the time-resolution in measuring the evaporation rate (~300 µs). Despite this limitation in precision, what is absolutely clear is that the rapid evaporation of volatile components from a volatile droplet is correlated to the amount of water (reproducibly) added to the droplet during the period of evaporation of the volatile organic component. However, these data suggest that at room temperature, droplets consisting entirely of a species whose ΔH_(vap)_ is > 61.7 kJ/mol would not entirely convert to water during evaporation; for example, although the ΔH_(vap)_ of 1-hexanol is 61.7 kJ/mol, its vapour pressure, and thus the mass flux from the droplet, is so low that evaporative cooling is insufficient to drive the condensation of water onto the evaporating droplet.

**Figure 6:**
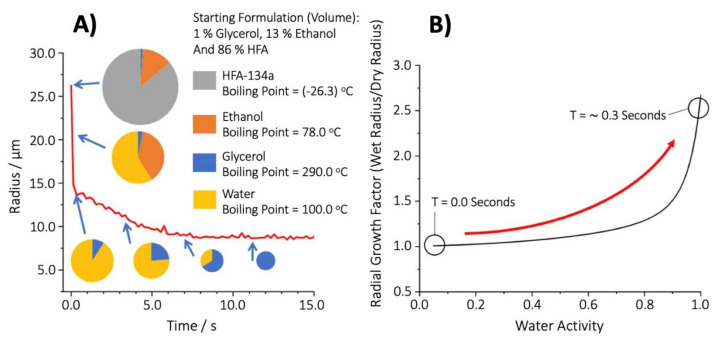
(**A**) Dynamic behaviour of a pharmaceutical aerosol for inhalation following aerosolization into 89% RH. The size and proportions of the inset pie charts are drawn to scale. (**B**) The change in water activity of the glycerol phase in an HFA/ethanol/glycerol droplet increases from 0 to 0.98 during the 0.3 s following droplet generation while the volatile HFA and ethanol evaporate. The final water activity is calculated from the change in size in the droplet in (**A**) using the hygroscopic growth curve of glycerol as shown. Note that the presence of the ethanol and HFA in the droplet will ensure that the droplet does not track the exact relationship observed in (**B**), but only arrives at the point indicated at 0.3 s following volatile loss and water condensation.

Indeed, the propensity for water to condense during the evaporation of volatile components for extremely highly volatile species, such as HFA, was experimentally confirmed. The evaporation profile of individual droplets originating from a pMDI starting formulation of HFA, ethanol and glycerol was directly measured (Figure 6A). Analogous to the series of volatile species considered previously (Figure 5), a dramatic shift from a composition of a volatile species to predominantly water was observed (the non-volatile glycerol remained in this case). Furthermore, the evaporation of both the HFA and ethanol correlated with a significant amount of water condensation onto the droplet. The uptake of water was then followed by the slow evaporation of water as the droplet, now a glycerol–water solution droplet, equilibrated to the surrounding conditions and ambient RH. This is the first ever direct measurement of the evaporation kinetics of an individual droplet originating from pMDI starting formulation of an appropriate size into conditions relevant to the lung.

As it pertains to the total and regional deposition in the lung, both the starting radius of the droplet and the water activity of the solvent within the droplet at the point of inhalation are critical. While the absolute radius is dependent on the initial droplet radius (physical mechanism), the propensity of the aerosol to grow during inhalation (which is entirely dependent on the evaporation dynamics prior to inhalation and the condensational dynamics during inhalation) is expected to be more constant across devices. There is no water present in the model pMDI formulation, so the droplets have an initial water activity of zero upon generation. However, the rapid cooling of the droplet, arising from the evaporation of the highly volatile HFA and ethanol, allows for the condensation of water onto the droplet and consequently the water activity of the droplet rapidly increases. The initial dry mass of glycerol in the pMDI formulation droplets can be calculated using the initial volume of the droplets and concentration of glycerol. By taking the droplet volume at the instance that all HFA and ethanol has evaporated, leaving a droplet comprising only of glycerol and water, and the estimated initial dry mass of glycerol (and converting them to the corresponding droplet radii), a growth factor of this ‘wet’ glycerol–water droplet can be calculated and thus the water activity determined. In doing so, it is shown that, whilst the overall droplet size is diminishing due to the evaporation of HFA and ethanol, the volume of water and water activity of the droplet is increasing from generation (0.00) to a maximum of ~0.98 at 0.3 s (the point at which the HFA and ethanol have fully evaporated) (Figure 6B). Based solely on the evaporative cooling from the evaporation of HFA and ethanol, the water activity of the aerosol produced from an pMDI inhaler is thus determined to be ~0.98 at the point of inhalation (Figure 6B). Following complete evaporation of the volatile components at 0.3 s, the droplet water activity begins to decrease from 0.98 as water evaporates as the droplet equilibrates to the ambient RH of 89%. The net effect on total and regional deposition of the dose as a result of droplets possessing a water activity of 0.98 upon inspiration is now discussed using a whole lung model wherein the detailed dynamics of the aerosol prior to and during inhalation are considered.

### 3.2. Effect of Water Uptake during HFA Evaporation on Total and Regional Dose in the Lung

**Figure 7:**
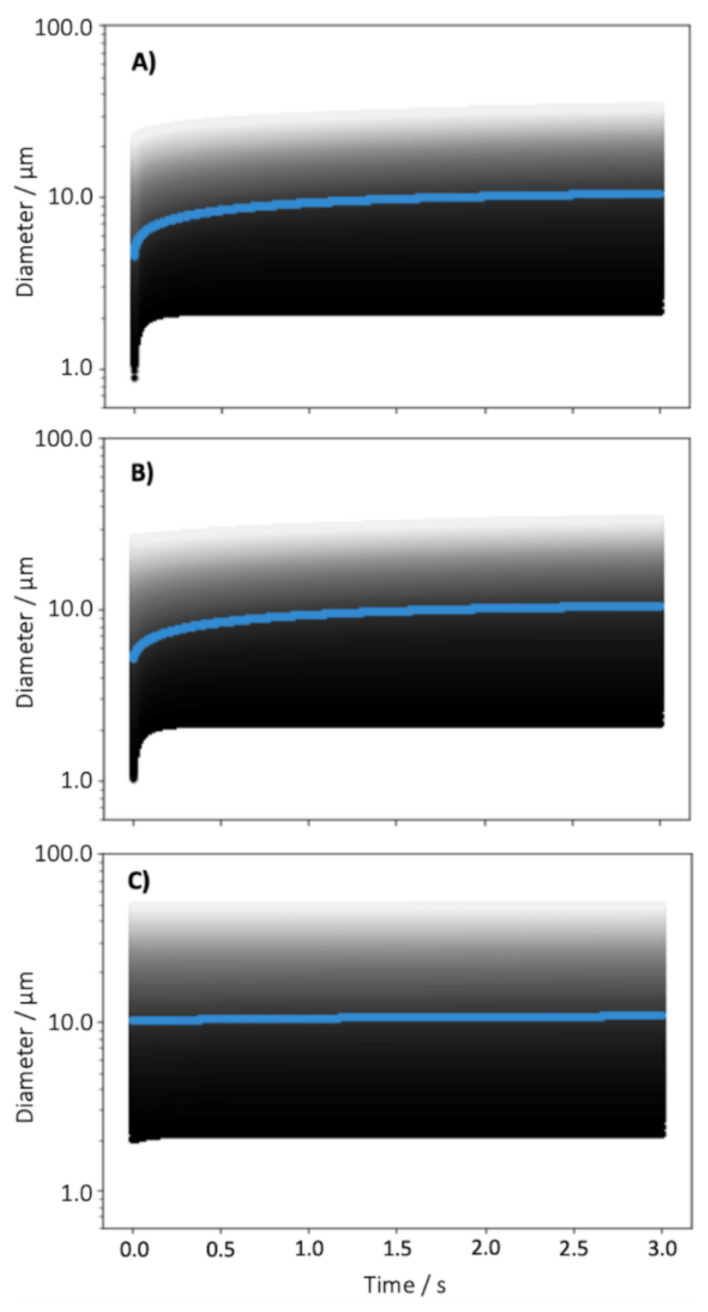
The condensational growth of a pre-equilibrated log normal droplet distribution (initial droplet diameter MMAD 10.5 µm, GSD 1.8 µm) upon inhalation. The three graphs illustrate the condensational growth during inhalation into the respiratory system (99.5 % RH). At *t* = 0 s, the aerosol droplet distribution has been pre-equilibrated (not shown) at 20% RH (**A**), 70% RH (**B**), or 98% RH (**C**). Each shade of grey (240 in total) follows the growth of an individual droplet within the model with a specific pre-equilibrated size.

A representative log-normal distribution was selected and the encompassing range of droplet sizes were simulated to equilibrate in environments of either 20, 70 or 98% RH within a spacer chamber before their condensational growth upon inhalation was calculated (Figure 7). As expected of droplets containing equivalent masses of glycerol, all three droplet distributions can be seen to tend towards the same final, equilibrated diameters in the 99.5% RH of the respiratory system. However, the different initial water activity and equilibrated droplet size distributions simulated display significant differences from each other in their rate and magnitude of condensational growth. The smallest starting droplet sizes, equilibrated in 20% RH, can be seen to grow the most drastically and rapidly, quickly catching and following the same growth curve as droplets equilibrated in 70% RH. This is in good accordance with the literature in which it is accepted that smaller droplets respond more quickly and substantially to changes in their environment, and that droplets of the same dry mass equilibrated at lower RH have a greater capacity for growth upon inhalation into the respiratory system [63]. In contrast, droplets equilibrated in 98% RH demonstrated only marginal growth upon inhalation, yielding less variability over the duration of a single breath, but were also significantly larger than those equilibrated in 20 or 70% RH at the point of inspiration. As droplet deposition in the respiratory system is a function of their aerodynamic diameter, these differences in the equilibrated diameters of the droplets and their relative growth are likely to impact their pulmonary deposition.

**Figure 8:**
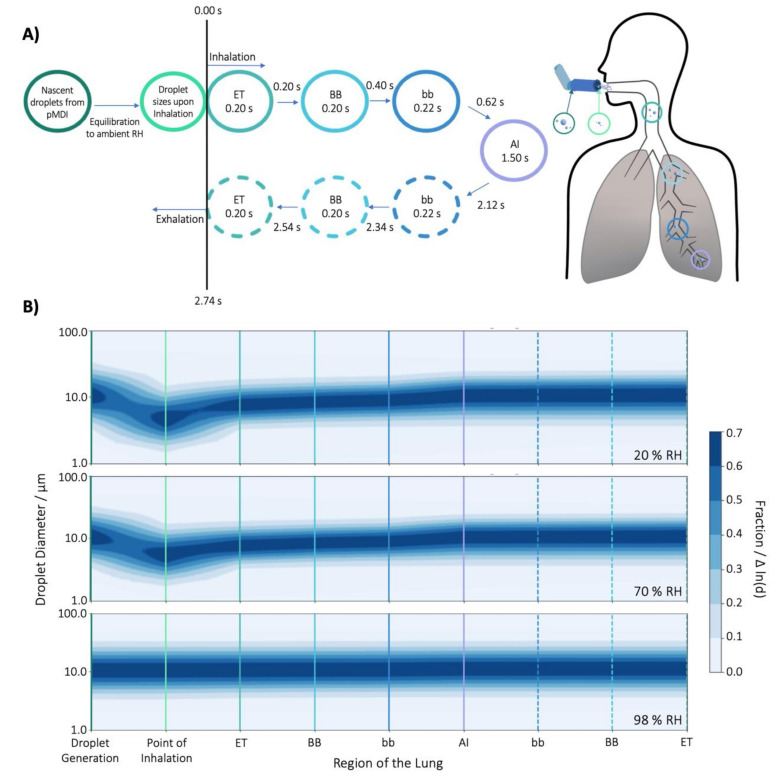
(**A**) The structure of the ICRP model with integrated semi-analytical hygroscopic growth model. Values inside the circles represent the residence time for the region. The cumulative time is presented above each at the exit point of a region. (**B**) The change in the log-normal distribution of glycerol–water solution droplet diameters at time corresponding to the ICRP model for three simulations in which the RH for equilibrium prior to inhalation is either 20, 70 or 98%. Fraction/Δ ln(d) is the fraction of the particle population lying between the interval between ln(d) and ln(dp) + d ln(dp) [56].

Spacer chambers are employed in pMDI therapies in order to reduce ballistic deposition in the oropharynx (allowing droplet evaporation and deceleration in the space), and to support a slower, more tidal inhalation compared to the use of pMDI without the spacer. The latter processes result in the preferential deposition of inhaled aerosol within the thoracic airways, and potentially deep lung deposition. In the current study, we examined whether the hygroscopic response of aerosol during inhalation from a spacer may also play a role in regulating pulmonary deposition profiles beyond that resulting from a controlled inhalation rate. Through modelling the droplet size evolution and extracting their diameters at time points corresponding to the moment the droplets transition from one ICRP region to the next (i.e., the cumulative residence time, Figure 8A), it is possible to see how the droplet size distribution fluctuates upon equilibration in ambient conditions in the spacer chamber and the conditions of the airways during a single breath (Figure 8B–D). Again, the results here show good agreement between the 20 and 70% RH environments, but significant differences between them and the droplets equilibrated at 98% RH. In the 20 and 70% RH cases, the particle sizes initially decrease upon generation as the population equilibrates to the surrounding RH, and then, at the instance of inhalation, begin to grow. This growth is fastest initially (in the ET region), when the difference between the droplet’s surface water activity and surrounding environment is greatest, but slows as the gradient between the two values diminishes until the final size distribution of the droplets stabilises upon departure from the AI region. This period of growth is also shown to be experienced uniquely by droplets of differing sizes within the distribution, with the smallest particles equilibrating most quickly, resulting in an initial narrowing of the size distribution. However, this contraction is momentary, and the distribution broadens again over time as the larger particles more slowly reach equilibrium, achieving a final distribution breadth upon expulsion from the AI region similar to that initially generated by the pMDI. In comparison, the distribution equilibrated in 98% RH remains static in its size distribution throughout inhalation, demonstrating only a very slight overall increase in particle size during residence within the ET region as the particles equilibrate from 98 to 99.5% RH, and no narrowing or broadening of the distribution. It should also be noted that the residence time in which growth may occur in the ET region is shorter other regions as the bronchiolar regions are summed and the AI region is represented as a single, combined time for inhalation and exhalation.

**Figure 9:**
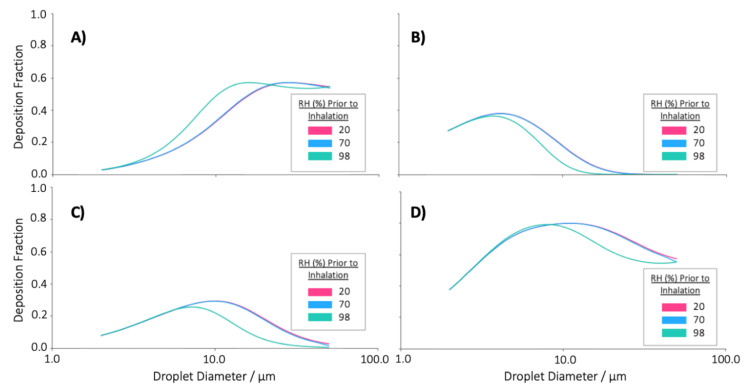
The regional and total deposition profiles of a log-normal distribution of droplets (emitted droplet size MMAD: 10.5 µm, GSD: 1.8), equilibrated in a spacer chamber at either 20, 70 or 98% RH prior to inhalation. (**A**) Deposition fraction in the ET region for different droplet diameters. (**B**) Deposition fraction in the BR region for different droplet diameters. (**C**) Deposition fraction in the AI region for different droplet diameters. (**D**) Total deposition fractions for different droplet diameters. N.B. The abscissa corresponds to the droplet diameters prior to inhalation and subsequent hygroscopic growth.

Particle deposition within the respiratory system has been shown to be highly dependent on aerosol particle size. As such, the perceived differences upon inhalation in the evolution of the droplet distributions initially equilibrated in 20, 70 or 98% RH suggest that particle deposition mechanisms act disparately upon the populations. Consequentially, it may be expected that the deposition profiles of the particles in the three environments would differ significantly, and indeed, from the ICPR modelling, we can see this to be the case (Figure 9).

It must be reiterated that the deposition of each equilibrated distribution was based on the same initial log-normal distribution of glycerol solution droplets generated from a pMDI. As such, the differences in deposition result from the changes the droplet plumes experienced upon equilibration in different RH (i.e., in the absence of RH effects, the size distribution upon inhalation, and thus the deposition profiles, would be equivalent). From Figure 9, there are minimal differences between the deposition fractions of particles equilibrated in 20% and 70% RH, with only a small shift in the deposition of the BR and AI region being observed for the larger droplets. Indeed, it is difficult to differentiate the two cases due to the high degree of conformity between them. In contrast, there is a significant difference between the deposition profiles of 20 and 70% RH and that of 98% RH. In the increased humidity, there is a substantial reduction in the BR, AI, and total deposition of particles with initial diameters greater than ~10 µm. This reduction in deposition in the lower lung regions is due to the droplet possessing larger sizes upon inhalation which result in increased impaction in the ET region. The increased ET deposition means that there are then fewer droplets available for deposition in the BR and AI regions, resulting in the reduced fraction presented.

**Figure 10:**
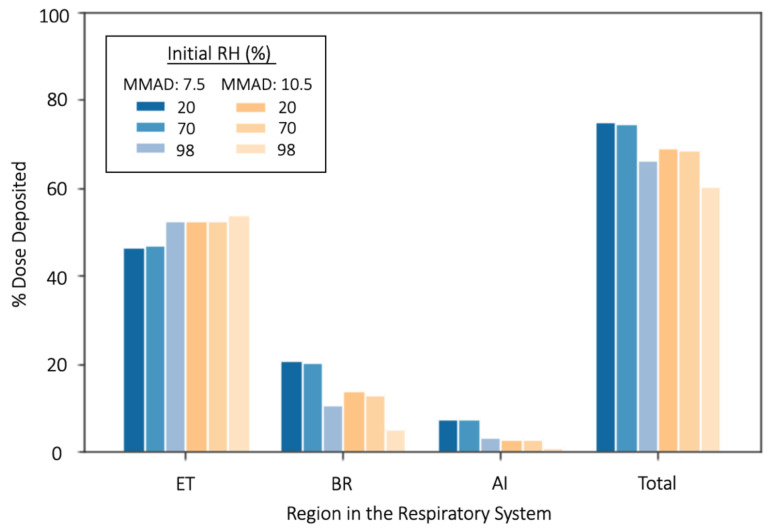
The regional and total dosage depositions in the respiratory system of two glycerol droplet distributions (MMAD: 7.5, 10.5, GSD: 1.8, 1.8) initially equilibrated in either 20, 70 or 98% RH.

By calculating the mass log-normal distribution of the pre-equilibrated droplets upon generation from the pMDI, it is possible to relate the deposition fraction to the deposited dose of glycerol from pMDI droplets in each of the ICRP regions. This estimate was undertaken for two size distributions taken from the literature (MMAD: 7.5 µm, 10.5 µm, GSD: 1.8, 1.8) [24], to investigate the effect that different initial pre-equilibration RHs and droplet diameters have on the deposited dose (Figure 10). From Figure 10, for all initial RH environments, the overall deposited dose is increased by approximately 5–7% as the size distribution is decreased from a MMAD of 10.5 to 7.5 µm. This is due to the increased deposition of droplets in the BR and AI region due to their smaller size. Furthermore, this smaller starting size demonstrates reduced ET deposition in comparison to the larger size distribution for all three RH environments. Whilst not reported here, dosage deposition calculations were also carried out for the two distribution sizes for a seated adult male and these results can be found in the Supplementary Materials Figure S1.

Both distributions demonstrate the same trends in deposition for the starting water activity distributions in each of the regions: in the ET region, deposition is seen to increase as the RH is increased, but for the other regions and total deposition, the inverse is true. In particular, a halving of the deposited dose in the BR and an almost complete elimination of droplet deposition in the AI region can be seen when going from 20 or 70% RH to 98% RH. Again, this can be accounted for by considering the impact of the increased RH on the size of the initial droplets at the point of inhalation. From Figure 7 and Figure 8, we can see that the droplets equilibrated into 98% RH have larger diameters at the instance of inhalation than those at lower RH because droplets in the 20 and 70% environments have undergone greater evaporation prior to inspiration. The resulting droplet population for 98% RH upon inhalation is therefore towards the upper end of respirable size and so a larger fraction is impacted in the ET airways and cannot deposit in the BR and AI regions. In contrast, the 20 and 70% equilibrated droplet distributions have diminished in diameter and so undergo less impaction in the ET region, but significantly increased deposition in the BR and Al region. However, it should be noted that the decrease in diameter may not result in increased deep deposition for all droplet distributions: for distributions with much smaller initial sizes, the reduction in diameter may mean they could have insufficient inertia or fall in a size range between those that are affected by either impaction and sedimentation or diffusion, and so be unable to deposit^48^. Ultimately though, this would also be dependent on their hygroscopicity and droplets that are small enough to pass through the ET region before growing to a size sufficient for deposition in the deep lung are being increasingly investigated for targeted drug delivery [64,65,66].

Overall, droplets equilibrated at 98% RH underwent significantly less hygroscopic growth upon inhalation and produced a dose deposition profile markedly different to that of droplets equilibrated in 20 or 70% RH for the size distributions considered, particularly in the deeper, BR and AI regions. These results have interesting implications for drug delivery to the lungs by pMDI. From the SPEL analysis of pMDI formulations, it has been calculated that water activity of the droplet is 0.98 upon inhalation. As such, the calculated growth and deposition profile for the droplets equilibrated in 98% RH allows us to begin to consider the potential depositional outcomes for pMDI formulations dispensed directly into the mouth. In contrast, the droplets equilibrated into 20% or 70% RH may be more representative of the inhalation of particles from pMDI with a spacer attached in either spring or autumn. There are obvious issues to this analogy, particularly regarding the differences in the deposition of the ballistic fraction experienced when using a spacer to a pMDI device alone. However, the marked variations in the deposition profiles of droplets equilibrated at 20, 70 and 98% provides compelling evidence for the need for further research into the effect that water condensation has on pMDI deposition as well as the difference in droplet dynamics for distributions dispensed into a spacer device versus the mouth.

## 4. Discussion

Assuming that the rate of growth and time taken to reach equilibrium is comparable to the length of a single breath, it is generally accepted that a lone, hygroscopic aerosol droplet would experience hygroscopic growth in the high humidities of the respiratory system. Yet, there is significant debate over whether there is sufficient water available within the lungs to facilitate the growth of a whole aerosol population to equilibrium. The consequence of this doubt has been a general oversight of the impact that hygroscopic growth has on the deposition of aerosols upon inhalation. However, in order to evaluate whether there is sufficient water present for population growth to occur, both the mass of water taken up by the aerosol plume and mass available in the lung must be quantified. The presented model calculates the mass flux of water for each droplet within a log-normal aerosol distribution, allowing the mass of water required for complete equilibration to be estimated by summation. For example, for a distribution of 100,000 droplets with an MMD of 10.5 µm and GSD of 1.8 (glycerol concentration of 10% *w*/*v*, ~24 µg glycerol), the total mass of water taken up by the plume in the length of a single, tidal inhalation from a spacer by a sitting, adult Caucasian female (2.74 s) is 12.4 ng. Further research is needed to ascertain the exact volume of water available and how quickly it is replenished in the respiratory system, especially for patients with pulmonary conditions, to allow the feasibility of the hygroscopic growth of aerosol populations to be accounted for.

## 5. Conclusions

For the optimisation of pMDI dose deposition and patient outcomes, it is imperative that the effect of the atomisation processes and secondary ageing of the aerosol droplet plume is fully understood. In this paper, we have presented the first direct measurement of the evaporation profile of an individual droplet originating from a pMDI starting formulation using the novel SPEL setup and, in doing so, have demonstrated unmistakable evidence for the condensational uptake of water by pMDI formulations following the rapid evaporation of propellant and ethanol co-solvent. Furthermore, the extent of water uptake was found to be directly correlated to the evaporation of volatile species, and the water activity of pMDI formulation at the point of inhalation, based solely on the evaporation of HFA and ethanol, was discerned to be 0.98. The effect of this initial water activity on pulmonary deposition was then explored using the ICRP model with an integrated hygroscopic growth treatment. In doing so, droplet distributions with an initial water activity of 0.98 were shown to display significantly less hygroscopic growth upon inhalation, as well as markedly different deposition profiles to those equilibrated to lower initial water activities at the instance of inspiration. There has been significant historical investigation of how the interactions between formulation properties (volatility and viscosity) and device design (spray orifice diameter) influence the aerosol size distribution for pMDIs to modulate respiratory deposition. The ability to tune the physical chemical interaction with water vapour after aerosol generation by controlling initial droplet size and composition may be a further approach for the optimization of deposition profiles of pMDI aerosols. Looking to the future, additional research into the precise amount of water added, as a function of initial droplet composition and the spray dynamics of droplets dispensed into a spacer or the mouth, is required.

## Figures and Tables

**Table 1:** Summary of abbreviations.

Abbreviation	Definition
pMDI	Pressurised metered dose inhaler
SPEL	Single Particle Electrodynamic Lung
HFA	Hydrofluoroalkane
ICRP	International Commission of Radiological Protection
RH	Relative humidity
MMAD	Mass median aerodynamic diameter
FPF	Fine particle fraction
CK-EDB	Comparative kinetic electrodynamic balance
MMD	Mass median diameter
GSD	Geometric standard deviation
ET	Extrathoracic region
BB	Bronchial region
Bb	Bronchiolar region
BR	Combined bronchial and bronchiolar region
AI	Alveolar interstitial region

**Table 2:** Variables used in modified ICRP model.

Symbol	Definition
*GF_r_*	Radial growth factor
*r(RH)*	Radius of wet droplet at a specific RH
*r(RH) =* 0	Radius of dry particle at 0% RH
*I*	Mass flux of water
*a*	Droplet radius
*S* *_∞_*	Saturation ratio of water in the gas phase away from droplet surface
*a* *_w_*	Water activity at the droplet surface
*R*	The gas constant
*_T∞_*	Temperature of the gas phase
*M*	Molar mass of water
*A*	Stefan flow correction factor
*D*	Diffusion coefficient of water in the gas phase
*p* *_v,∞_*	Saturation vapour pressure of water
*L*	Latent heat of vaporisation of water
*K*	Thermal conductivity of the carrier gas
*β* *_M_*	Fuchs–Sutugin transitional correction factor for mass transfer
*β* *_T_*	Fuchs–Sutugin transitional correction factor for heat transfer
*Sh*	Sherwood number
*η*	Deposition efficiency (also filtration efficiency)
*a* *_ICRP_*	Constant used to calculate the deposition efficiency in the ICRP model
*R* *_ICRP_*	Function of droplet diameter and airflow used to calculate the deposition efficiency in the ICRP model
*p*	Constant used to calculate the deposition efficiency in the ICRP model
*η* *_Total_*	Total deposition efficiency
*η* *_ae_*	Aerodynamic deposition efficiency
*η* *_th_*	Thermodynamic deposition efficiency
*DE*	Deposition fraction
*η* *_j_*	Total deposition efficiency for region *j*
*j*	Region of the ICRP model (also respiratory system)
*φ* *_j_*	Volumetric fraction for region *j*
*N*	Number of regions

## Data Availability

Data are available at the University of Bristol data repository, data.bris, doi:10.5523/bris.2cym5ti2vpfyp1zoydiqqoj7ho.

## Supplementary Materials

The following are available online at https://www.mdpi.com/article/10.3390/pharmaceutics13070941/s1, Figure S1: The regional and total dosage depositions in the respiratory system of two glycerol droplet distributions (MMAD: 7.5, 10. 5, GSD: 1.8, 1.8) initially equilibrated in either 20, 70 or 98% RH. ICRP simulation set to an adult Caucasian male who was seated, Table S1: Algebraic Expressions for Aerodynamic and Thermodynamic Deposition by Mouth Breathing in the ICRP Model, Table S2: Physiological Parameters used in the ICRP Model.
