# Supplementary material for: Water Uptake by Evaporating pMDI Aerosol Prior to Inhalation Affects Both Regional and Total Deposition in the Respiratory System
Source: Pharmaceutics. 2021 Jun 24;13(7):941. doi: 10.3390/pharmaceutics13070941 (PMC8309010; doi:10.3390/pharmaceutics13070941)
Supplement: Supplementary file 1 [file pharmaceutics-13-00941-s001.zip › pharmaceutics-1203292-supplementary.pdf]

pharmaceutics

MDPI

Supplementary Materials: Water Uptake by Evaporating pMDI Aerosol Prior to Inhalation Affects Both Regional and Total Deposition in the Respiratory System

Victoria Legh-Land, Allen E. Haddrell, David Lewis, Darragh Murnane and Jonathan P. Reid

Table S1. Algebraic Expressions for Aerodynamic and Thermodynamic Deposition by Mouth Breathing in the ICRP Model (adapted from ICRP 66. *Annals of the ICRP*, 1994).

		Aerodynamic			Thermodynamic			
		$a_{(ICRP)}$	$R_{(ICRP)}$	p	$a_{(ICRP)}$	$R_{(ICRP)}$	p	
Inhalation	ET*	1.1×10^{-4}	$d_{ae}^2(\dot{V}SF_t^3)^{0.6}(V_T SF_t^2)^{-0.2}$	1.4	9.0	$D(\dot{V} SF_t)^{-1/4}$	0.5	1
	BB	4.08×10^{-6}	$d_{ae}^2 \dot{V} SF_t^{2.3}$	1.152	$22.02 SF_t^{1.24} \Psi_{th}$	Dt_B	0.6391	$1-(V_D(ET)/V_T)$
	bb	0.1147	$(0.056 + t_b^{1.5}) \times d_{ae}^{t_b^{-0.25}}$	1.173	$-76.8 + 167 SF_b^{0.65}$	Dt_b	0.5676	$1-[(V_D(ET)+V_D(BB))/V_T]$
	AI	$0.146 \times SF_A^{0.98}$	$d_{ae}^2 t_A$	0.6495	$170 + 103 SF_A^{2.13}$	Dt_A	0.6101	$1-[(V_D(ET)+V_D(BB)+V_D(bb))/V_T]$
Exhalation	bb	0.1147	$(0.056 + t_b^{1.5}) \times d_{ae}^{t_b^{-0.25}}$	1.173	$-76.8 + 167 SF_b^{0.65}$	Dt_b	0.5676	$1-[(V_D(ET)+V_D(BB))/V_T]$
	BB	2.04×10^{-6}	$d_{ae}^2 \dot{V} SF_t^{2.3}$	1.152	$22.02 SF_t^{1.24} \Psi_{th}$	Dt_B	0.6391	$1-(V_D(ET)/V_T)$
	ET*	1.1×10^{-4}	$d_{ae}^2(\dot{V}SF_t^3)^{0.6}(V_T SF_t^2)^{-0.2}$	1.4	9.0	$D(\dot{V} SF_t)^{-1/4}$	0.5	1

*For this region alternative equation is used to determine the aerodynamic Regional Deposition Efficiency, η_{ae}:.

$\eta_{ae} = 1-1/(a_{(ICRP)}R_{(ICRP)}^{p(ICRP)}+1)$.

d_{ae} Aerodynamic diameter

SF Scaling factor to account for subject's size (see Table S-2)

D Diffusion constant

Ψ_{th} Empirical correction factor to account for the turbulent airflow in the initial generations of the tracheal-bronchial tree

t Residence time (s) in a given region (region indicated by subscript).

Table S2. Physiological Parameters used in the ICRP Model (adapted from ICRP 66. *Annals of the ICRP*, 1994).

Parameter	Female (Sitting)	Male (Sitting)
Functional Residual Capacity (FRC) (mL)	2681	3301
Extrathoracic Dead Space (V_D(ET)) (mL)	40	50
Bronchi and Tracheal Dead Space (V_D(BB)) (mL)	40	49
Bronchiole Dead Space (V_D(bb)) (mL)	44	47
Tracheal Scale Factor (SF_t)	1.08	1.00
Bronchiolar Scale Factor (SF_b)	1.04	1.00
Respiratory Bronchiole Scale Factor (SF_a)	1.07	1.00
Tidal Volume (V_T) (mL)	464	750
Volumetric Flow Rate ($\dot{V}$) (mL s^{-1})	217	300

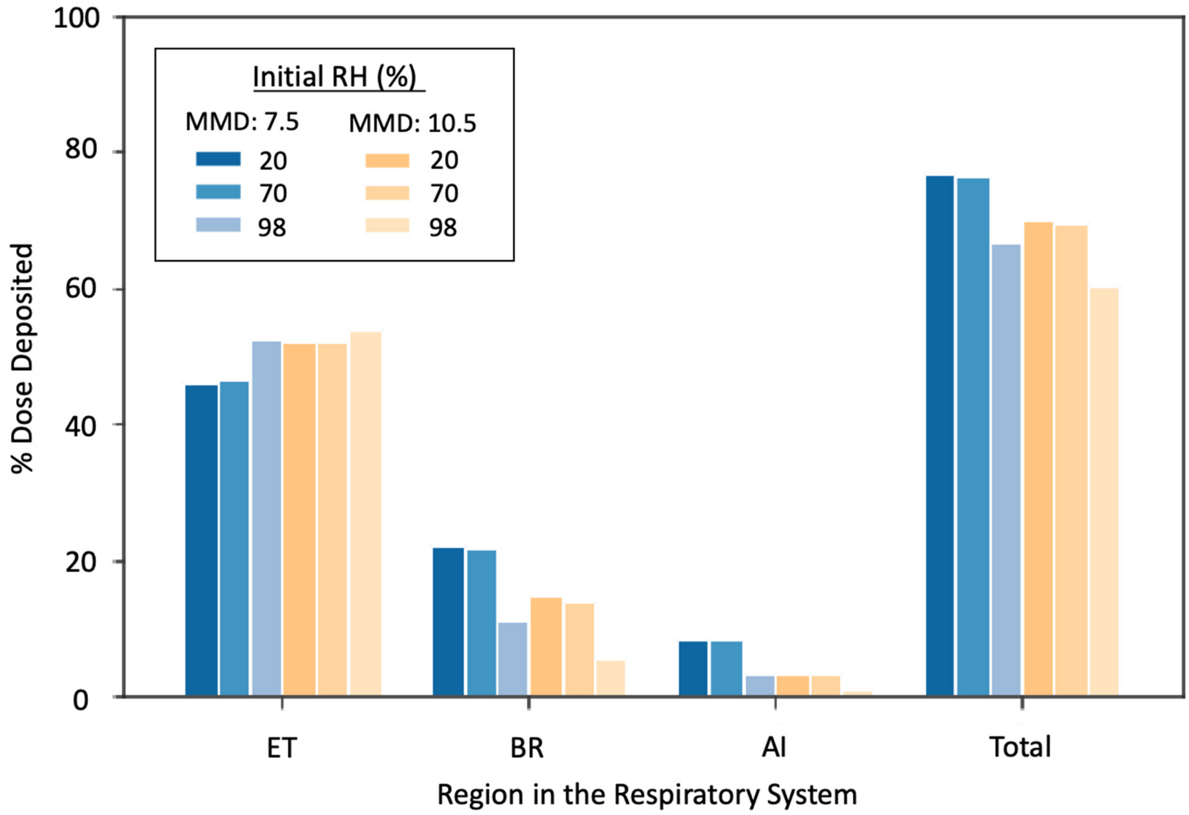

Figure S1. The regional and total dosage depositions in the respiratory system of two glycerol droplet distributions (MMAD: 7.5, 10. 5, GSD: 1.8, 1.8) initially equilibrated in either 20, 70 or 98 % RH. ICRP simulation set to an adult Caucasian male who was seated.
